# High-dose cytarabine monotherapy is superior to standard-dose cytarabine- based multiagent sequential treatment cycle for consolidation treatment in adult (14-59 years) AML patients according to European Leukemia Net 2022 risk stratification

**DOI:** 10.3389/fonc.2022.1070588

**Published:** 2023-01-16

**Authors:** Xiaoyu Wang, Dan Liu, Erling Chen, Li Wang, Na Zhao, Li Zhou, Juan Tong, Lei Xue, Lei Zhang, Liangquan Geng, Baolin Tang, Huilan Liu, Xin Liu, Changcheng Zheng

**Affiliations:** ^1^ Department of Hematology, Anhui Provincial Hospital, Anhui Medical University, Hefei, China; ^2^ Department of Hematology, The Affiliated Provincial Hospital, Wannan Medical College, Wuhu, China; ^3^ Department of Hematology, The First Affiliated Hospital of USTC, Division of Life Sciences and Medicine, University of Science and Technology of China, Hefei, China

**Keywords:** acute myeloid leukemia, 2022 European Leukemia Net, high-dose cytarabine, multiagent sequential chemotherapy, consolidation

## Abstract

**Introduction:**

We firstly investigate based on 2022 European Leukemia Net (ELN) risk stratification, whether standard-dose cytarabine based multiagent sequential chemotherapy (SDMSC) is more beneficial than high-dose cytarabine (HDAC) monotherapy in consolidation for the survival of adult acute myeloid leukemia (AML) patients.

**Methods:**

One hundred and eighty-three AML patients with complete remission (CR) were evaluated.

**Results and discussion:**

The 3-year relapse rate was 33.4% in the HDAC group and 50.5% in the SDMSC group (*p*=0.066). The 3-year overall survival (OS) and event-free survival (EFS) rates in the HDAC group (69.2%, 60.7%) were significantly higher than that in the SDMSC group (50.8%, 42.1%) (*p*=0.025, 0.019). For patients in the intermediate risk group, the 3-year OS and EFS rates in the HDAC group (72.5%, 56.7%) were higher than that in the SDMSC group (49.1%, 38.0%) (*p*=0.028, 0.093). This study indicates that for young adult AML patients, HDAC consolidation achieves a higher long-term survival than SDMSC, especially for patients in the intermediate-risk group according to the 2022 ELN risk stratification.

## Introduction

The standard treatment of acute myeloid leukemia (AML) consists of one or two cycles of chemotherapy to induce complete remission (CR) followed by post-remission treatment to improve the duration of long-term remission. Only about 35 to 40% of CR patients can achieve long-term survival without disease recurrence (1), and post-remission therapy is mandatory to prevent relapse. Multiple cycles of high-dose cytarabine (HDAC) have been commonly used as standard consolidation treatment for AML patients who achieved CR in Europe and the United States (2). However, sequential multiagent chemotherapy using non–cross-resistant agents were also commonly used in Asian countries such as Japan; the JALSG AML 201 study (3) demonstrated that the multiagent chemotherapy regimen is as effective as HDAC regimen for consolidation, however, the HDAC regimen was accompanied with more severe and longerlasting neutropenia leading to more frequent infectious events.

To further investigate based on 2022 ELN risk stratification (4), whether standard-dose cytarabine based multiagent sequential chemotherapy (SDMSC) is more beneficial than HDAC monotherapy in consolidation for the survival of adult AML patients. We retrospectively analyzed the clinical features of newly diagnosed AML patients under 60 years from June 2015 to December 2020 in our center who achieved CR after the first induction therapy with IA (3 + 7) regimen, and compared the efficacy of SDMSC with HDAC regimens for consolidation therapy, focusing on the disease relapse and long-term survival (follow-up to June 2022).

## Patients and methods

### Patients

**Figure 1:**
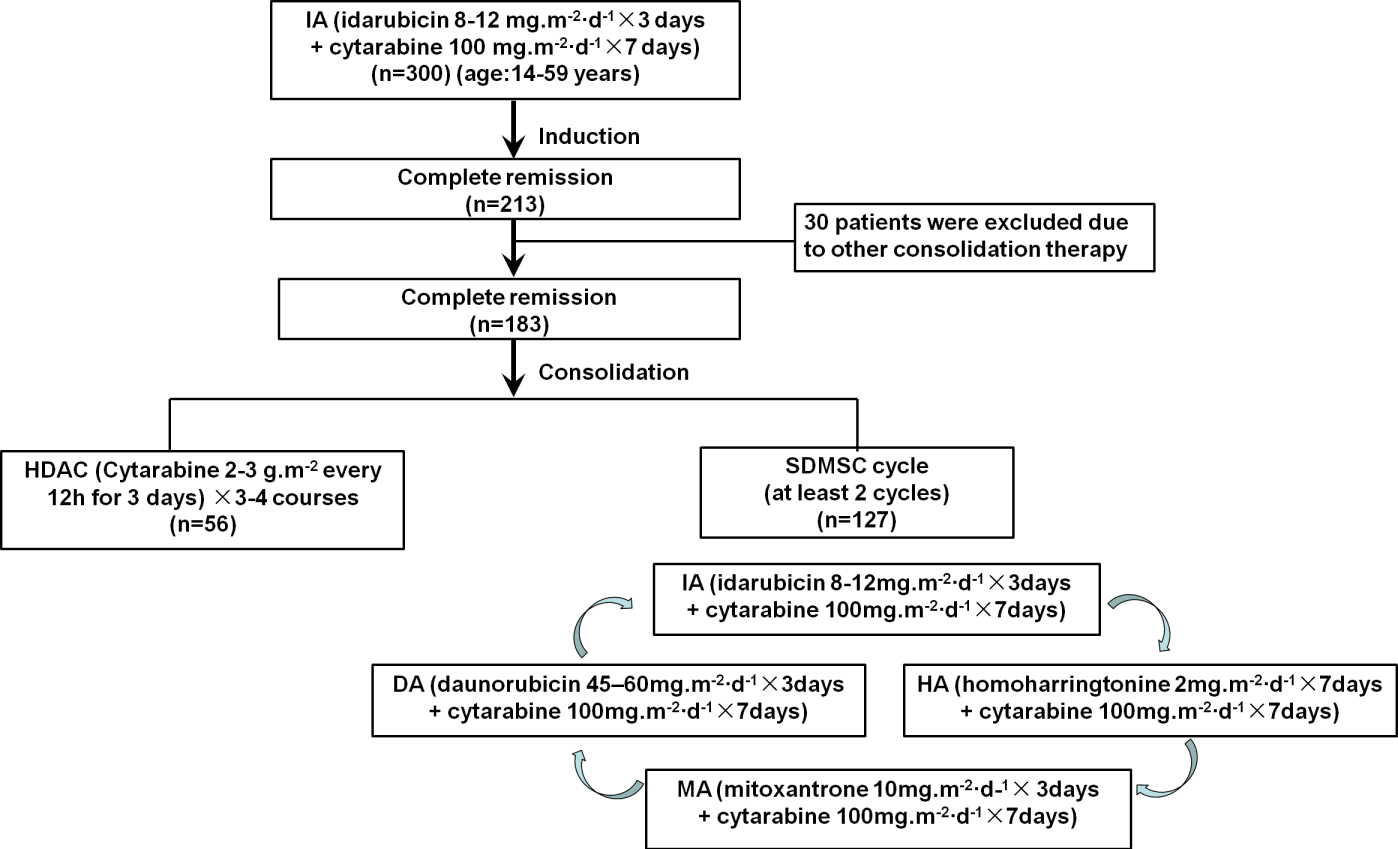
Scheme of the study protocol.

This study retrospectively analyzed 213 patients with newly diagnosed AML who achieved CR after first induction therapy with the IA (3 + 7) regimen at the First Affiliated Hospital of the University of Science and Technology of China (Anhui Provincial Hospital) from June 2015 to December 2020. The screening criteria were 14 years or older and 59 years or younger, complete remission after the first induction with the IA (3 + 7) regimen; and patients with acute promyelocytic leukemia were excluded. Among the patients studied, 30 patients were excluded due to these patients received other consolidation therapy. The remaining 183 patients were divided into two groups, including 127 patients received SDMSC and the other 56 patients received HDAC (**Figure 1**). This study protocol was approved by the ethics committee of Anhui Provincial Hospital and was conducted in accordance with the Declaration of Helsinki (2022-RE-329).

### Treatment

All patients in CR after induction therapy with IA (3 + 7) (idarubicin 8-12mg/m^2^/day for 3 days + cytarabine 100mg/m^2^/day for 7 days) followed by consolidation therapy, either 3-4 courses of HDAC (2-3g/m^2^ every 12h for 3 days) or SDMSC. SDMSC consisted of at least 2 cycles of multiagent sequential chemotherapy, and each cycle were conducted in the following order: IA (idarubicin 8-12 mg/m2/day for 3 days + cytarabine 100 mg/m2/day for 7 days), followed by HA (homoharringtonine 2 mg/m2/day for 7 days + cytarabine 100 mg/m2/day for 7 days), MA(mitoxantrone 10mg/m2/day for 3 days + cytarabine 100 mg/m2/day for 7 days), DA (daunorubicin 45-60mg/m2/day for 3 days + cytarabine 100 mg/m2/day for 7 days). Patients at intermediate or adverse risk are eligible for further evaluation of hematopoietic stem cell transplantation (HSCT).

### Definitions and statistical analysis

Risk- stratification was derived from the 2022 European Leukemia Net (ELN) recommendations on diagnosis and management of AML in adults (4). The definition of CR, relapse, overall survival (OS) and event-free survival (EFS) were defined as reported elsewhere (5). Cumulative incidence of relapse (CIR) was defined as time from remission to relapse for all patients who achieved CR, and patients who died without relapse were considered competing causes of failure. Patient-, disease-, and transplant-related variables were measured using chi-square test (categorical variables), Mann-Whitney U-test (continuous variables). Relapse was generated by the cumulative-incidence function method, taking competing risks into account. The probabilities of OS and EFS were generated by the Kaplan-Meier method. R statistical software was used for statistical analysis (R Foundation for Statistical Computing). Differences were considered statistically significant at *p*< 0.05.

## Results

### Clinical characteristics

One hundred and eighty-three CR patients were evaluated. Among them, 56 patients received treatment of HDAC (HDAC group), and 127 patients received treatment of SDMSC (SDMSC group). There were no significant differences in age, sex, 2022 ELN risk stratification, underlying disease, white blood cell count at first diagnosis, minimal residual disease (MRD) after induction, MRD prior HSCT, and molecular abnormalities between two consolidation groups (**Table 1**).

**Table 1:** Baseline clinical characteristics of patients.

Characteristics	SDMSC (n=127)	HDAC (n=56)	*p* -value
**Age (years), Median (range)**	44 (16-58)	43 (14-58)	*p*=0.426
**Sex ratio, M/F, no. (%)**	58/69 (45.7/54.3)	28/28 (50.0/50.0)	*p*=0.632
**ELN risk assessment, no. (%)**			*p*=0.610
Favorable	40 (31.5)	18 (32.1)	
Intermediate	61 (48.0)	30 (53.6)	
Adverse	26 (20.5)	8 (14.3)	
**Underlying disease, no. (%)**			*p*=0.701
Hypertension	11 (8.7)	4 (7.1)	
Diabetes	8 (6.3)	2 (3.6)	
Rheumatism	4 (3.1)	1 (1.8)	
Virus hepatitis	5 (3.9)	1 (1.8)	
Malignant tumor	1 (0.8)	1 (1.8)	
Pregancy	4 (3.1)	2 (3.6)	
Myeloid sarcoma	0	1 (1.8)	
Stoke	1 (0.8)	1 (1.8)	
Arhythmia	1 (0.8)	1 (1.8)	
Parkinson’s disease	1 (0.8)	0	
Hyperthyroidism	2 (1.6)	0	
Hypothyroidism	1 (0.8)	1 (1.8)	
Pulmonary embolism	0	1 (1.8)	
**WBC at first diagnosis (×10^9^/L), Median (range)**	12.76 (0.96-477.26)	10.11 (1.12-383.22)	*p*=0.790
**Molecular biology**			*p*=0.073
CEBPA mutation, no. (%)	24 (18.9)	9 (16.1)	
FLT3-ITD mutation, no. (%)	8 (6.3)	1 (1.8)	
NPM1 and FLT3-ITD mutation, no. (%)	2 (1.6)	5 (8.9)	
NPM1 mutation, no. (%)	11 (8.7)	8 (14.3)	
Negative detection, no. (%)	44 (34.6)	13 (23.2)	
Others	13 (10.2)	10 (17.9)	
Not available, no. (%)	25 (19.7)	10 (17.9)	
**MRD after induction**			*p*=0.704
** Negative, no. (%)**	80 (63.0)	29 (51.8)	
** Positive, no. (%)**	35 (27.6)	15 (26.8)	
** Not available, no. (%)**	12 (9.4)	12 (21.4)	
**Transplantation, no. (%)**	29 (22.8)	20 (35.7)	*p*=0.102
**MRD before transplantation**			*p=0.552*
** Negative, no. (%)**	17 (58.6)	8 (40.0)	
** Positive, no. (%)**	4 (13.8)	0	
** Not available, no. (%)**	8 (27.6)	12 (60.0)	
**Relapse, no. (%)**	59 (46.5)	18 (32.1)	*p*=0.076
**Follow-up time (months), median (range)**	26 (2-84)	30 (5-74)	*p*=0.241

### Overall results

**Figure 2:**
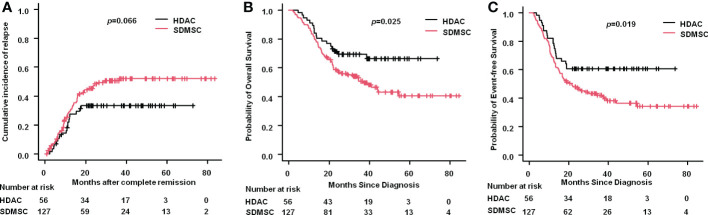
Relapse and survival in patients of aml. Cumulative incidence of relapse for the whole cohort **(A)**. Probability of overall survival for the whole cohort **(B)**. Probability of event-free survival for the whole cohort **(C)**.

The 3-year CIR was 33.4% (95% CI:22.5%-47.7%) in the HDAC group and 50.5% (95% CI:41.6%-60.2%) in the SDMSC group (*p*=0.066) (**Figure 2A**). The 3-year OS rate in the HDAC group was significantly higher than that in the SDMSC group, 69.2% (95% CI:55.1%-79.6%) vs 50.8% (95% CI:41.4%-59.4%), respectively(*p*=0.025) (**Figure 2B**). The 3-year-EFS in the HDAC group was significantly higher than that in the SDMSC group, 60.7% (95% CI:46.7%-72.1%) vs 42.1% (95% CI:33.3%-50.7%) (*p*=0.019) (**Figure 2C**).

### Results according to 2022 ELN risk stratification

**Figure 3:**
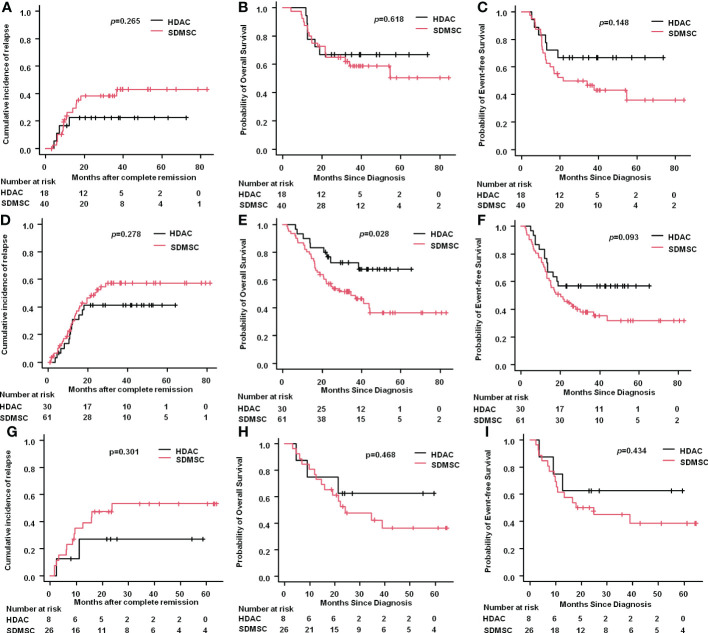
Results according to 2022 ELN risk stratification. Cumulative incidence of relapse for favourable-risk patients **(A)**. Probability of overall survival for favourable-risk patients **(B)**. Probability of event-free survival for favourable-risk patients **(C)**. Cumulative incidence of relapse for intermediate-risk patients **(D)**. Probability of overall survival for intermediate-risk patients **(E)**. Probability of event-free survival for intermediate-risk patients **(F)**. Cumulative incidence of relapse for adverse-risk patients **(G)**. Probability of overall survival for adverse-risk patients **(H)**. Probability of event-free survival for adverse-risk patients **(I)**.

For patients in the favorable risk group, the 3-year CIR in the HDAC group was 22.6% (95%CI: 9.1%-49.7%), which was similar to that in the SDMSC group, 38.5% (95%CI:24.8%-56.3%) (*p*=0.265) (**Figure 3A**). The 3-year OS rate of patients in the HDAC group was 66.7% (95%CI:40.4%-83.4%) and in the SDMSC group was 58.8% (95%CI:41.7%-72.5%) (p=0.618) (**Figure 3B**). The 3-year EFS rate in the HDAC group was 66.7%(95%CI:40.4%-83.4%), which was slightly higher than that in the SDMSC group 46.8%(95%CI:30.8%-61.4%) (*p*=0.148) (**Figure 3C**).

For patients in the intermediate risk group, the 3-year CIR was 41.2% (95%CI:25.9%-61.1%) in the HDAC group and 57.3%(95%CI:44.4%-70.9%) in the SDMSC group (*p*=0.278) (**Figure 3D**). The 3-year OS rate of patients in the HDAC group was 72.5%(95%CI:52.3%-85.3%), which was significantly higher than that in the SDMSC group 49.1%(95%CI:35.5%-61.3%) (*p*=0.028) (**Figure 3E**). The 3-year EFS rate was 56.7%(95%CI:37.3%-72.1%) in the HDAC group, which was slightly higher than that in the SDMSC group 38.0%(95%CI:25.7%-50.3%) (*p*=0.093) (**Figure 3F**).

For patients in the adverse-risk group, there were no significant differences between patients in the HDAC group and in the SDMSC group, in terms of 3-year CIR [27.1%(95%CI:7.5%-72.4%) vs 53.1%(95%CI:34.7%-74.0%), p=0.301] (**Figure 3G**), 3-year OS rate[62.5%(95%CI:22.9%-86.1%) vs 42.1%(95%CI:22.4%-61.2%), p=0.468] (**Figure 3H**), and 3-year EFS rate[62.5%(95%CI:22.9%-86.1%) vs 45%(95%CI:25.2%-63.0%), p=0.434] (**Figure 3I**).

### Impact of transplantation

**Figure 4:**
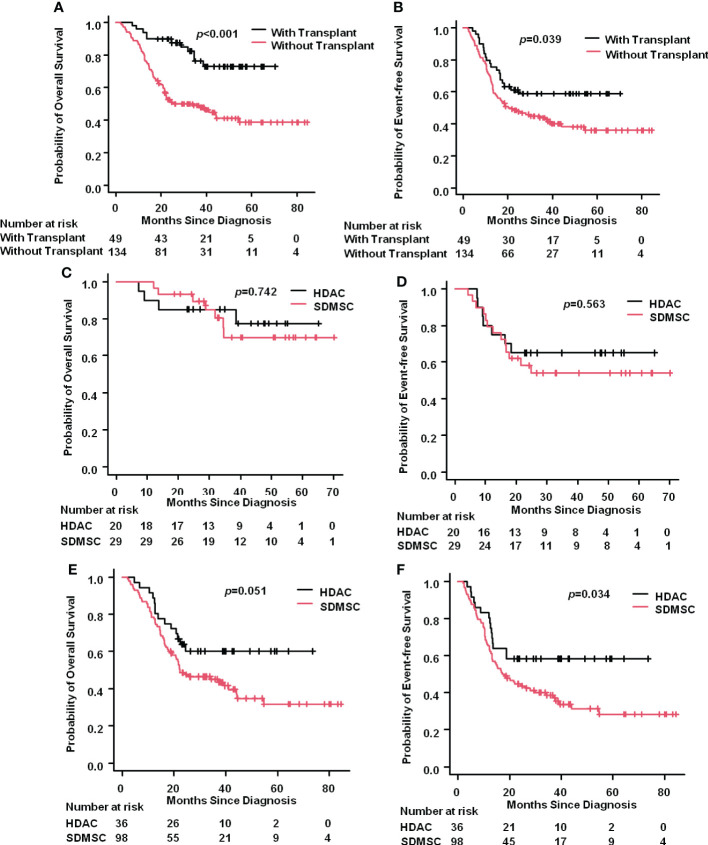
Impact of transplantation. The 3-year OS rate of AML patients with or without transplantation **(A)**. The 3-year EFS rate of AML patients with or without transplantation **(B)**. For the transplantation patients, the 3-year OS rate in the HDAC group and in the SDMSC group **(C)**, and the 3-year EFS rate in the HDAC group and in the SDMSC group **(D)**. For patients without transplantation, the 3-year OS rate in the HDAC group and in the SDMSC group **(E)**, and the 3-year EFS rate in the HDAC group and in the SDMSC group **(F)**.

Allogenetic hematopoietic stem cell transplantation (HSCT) was performed in 20(35.7%) and 29(22.8%) patients in the HDAC and SDMSC groups, respectively. Among them, 15 and 19 patients in the HDAC and SDMSC groups, respectively, underwent cord blood transplantation, 5 and 10 patients underwent haploidentical HSCT(*p*=0.542). The 3-year OS rate of the transplantation patients reached 76.2%(95%CI:59.7%-86.6%), while the 3-year OS rate of patients without transplantation was only 49.0% (95% CI:40.1%– 57.3%) (*p*<0.001) (**Figure 4A**). Similarly, the 3-year EFS rate of the transplantation patients was 58.6%(95%CI:43.4%-71.1%), which was higher than that patients without transplantation 43.8%(95%CI:35.1%-52.2%) (*p*=0.039) (**Figure 4B**).

For the transplantation patients, the 3-year OS rate in the HDAC group and in the SDMSC group were 77.3%(95%CI:49.0%-91.1%) and 69.8%(95%CI:46.0%-84.6%) (*p*=0.742), respectively (**Figure 4C**). And the 3-year EFS rate in the HDAC group and in the SDMSC group were 65.0%(95%CI:40.3%-81.5%) and 54.0%(95%CI:34.1%-70.3%) (*p*=0.563), respectively (**Figure 4D**).

For patients without transplantation, the 3-year OS rate in the HDAC group was 60.1%(95%CI:41.9%-74.2%), which was slightly higher than that in the SDMSC group 45.0%(95%CI:34.8%-54.7%) (*p*=0.051) (**Figure 4E**). The 3-year EFS rate was 58.3% (95% CI:40.7%-72.4%) in the HDAC group, which was significantly higher than that of 38.7% (95% CI:28.9%-48.4%) in the SDMSC group (*p*=0.034) (**Figure 4F**).

## Discussion

This retrospective study demonstrated that for young adult AML patients, HDAC (2-3 g/m2 every 12 hours on d1-3) achieves a higher long-term survival than SDMSC regimens based on standard-dose cytarabine (cytarabine 100 mg.m-2·d-1×7 days), especially for patients in the intermediate-risk group according to the 2022 ELN risk stratification.

Regarding the consolidation strategies, HDAC was commonly used in countries such as the United States since the landmark of Cancer and Leukemia Group B-8525 trial (CALGB-8525) was reported (6). This study indicated a significant dose-dependent effect of cytarabine in the postremission treatment for AML. Patients 60 years of age or younger who received HDAC (3 g/m2 every 12 hours on D1, D3, and D5) had lower relapse and higher long-term survival rates than patients who received lower doses of cytarabine (100 mg/m2 on D1-5 or 400 mg/m2 on D1-5). However, the CALGB-8525 trial only demonstrated that HDAC alone was superior to lower-dose cytarabine alone and did not assess the effects of combination regimens in consolidation. The Acute Leukemia French Association Group trial (ALFA-9802) (7) compared HDAC (3 g/m^2^) consolidation regimen with a timed-sequential consolidation regimen consisting of etoposide, mitoxantrone, and cytarabine (500 mg/m^2^, d1-d5) and showed the HDAC regimen was more beneficial for patient survival.

However, not all studies support that HDAC consolidation is superior to multi-agent combination regimens. The JALSG AML201 trial (3) demonstrated that the lower-dose cytarabine (200 mg/m2 d1-d5) regimen combined with mitoxantrone, daunorubicin, aclarubicin, or etoposide was as effective as HDAC (2 g/m2 twice daily for 5 days) in postremission consolidation, and recommended that the conventional multiagent chemotherapy may be suitable for the AML patients in intermediate or adverse cytogenetic risk groups. The Cancer and Leukemia Group B 9222 trial (CALGB-9222) (8) showed that sequential multiagent chemotherapy had similar disease-free survival to HDAC for post-remission treatment of adults under 60 years of age. The Medical Research Council AML15 Trial (9) indicated that multiagent consolidiation regimens [amsacrine, cytarabine, and etoposide (MACE) followed by mitoxantrone and cytarabine (MidAC)] achieved similar results to HDAC for patients with favorable and intermediate risk but superior in patients with high-risk disease, although it was associated with more hematologic toxicity.

This study aimed to explore whether the SDMSC using non–cross-resistant agents might improve long-term survival. First, in this study, the 3-year OS rate and EFS rate of patients in the HDAC group (69.2% and 60.7%) were significantly higher than that of SDMSC group (50.8% and 42.1%), especially for patients in the intermediate risk group (72.5% and 56.7% in the HDAC group vs 49.1% and 38.0% in the SDMSC group). These results indicated that HDAC (2-3 g/m2 every 12 hours on d1-3) with 3~4 courses is the preferred consolidation regimen for young adult AML patients. In addition, we investigated whether patients with allo-HSCT had an OS or EFS benefit in comparison to those without allo-HSCT. As previously reported (10, 11), among patients at intermediate or adverse risk, an allogeneic transplant had an improved long-term survival, suggesting a positive impact of allo-HSCT in these patients. Although the most effective post-remission treatment is allo-HSCT, it is not available to all patients with intermediate or high-risk disease because of high rates of treatment-related complications and lacking suitable donors. Interestingly, for patients with transplantation, 3-year OS or EFS in the HDAC group was similar with that in the SDMSC group, suggesting that for patients undergoing transplantation, there was no survival differences for either receiving HDAC or SDMSC regimens prior to transplantation. However, for patients who do not receive transplantation, 3-year OS and EFS in the HDAC group were significantly higher than that in the SDMSC group, suggesting that HDAC consolidation is the preferred regimen for AML patients who have no opportunity to receive allo-HSCT.

Finally, to our knowledge, our study is the first to investigate the impact of consolidation treatment options for AML according to the ELN-2022 risk stratification system. Recently, the ELN published the revised risk stratification system for AML (ELN-2022) and several modifications have been made (4); AML with *FLT3*-ITD are now categorized in the intermediate-risk group, AML with myelodysplasia-related gene mutations is now categorized in the adverse-risk group, the presence of adverse-risk cytogenetic abnormalities in *NPM1*-mutated AML now defines adverse risk, etc. In this study, we demonstrated that for patients in the intermediate risk group, the 3-year OS rate of patients in the HDAC group was significantly higher than that in the SDMSC group (72.5% vs 49.1%, p=0.028); however, for patients in the favorable risk and adverse-risk groups, there were no significant differences between patients in the HDAC group and in the SDMSC group, in terms of 3-year CIR, OS and EFS.

In summary, this study indicates that for young adult AML patients, HDAC consolidation achieves a higher long-term survival than SDMSC, especially for patients in the intermediate-risk group according to the 2022 ELN risk stratification. Allo-HSCT is preferred for selected patients with intermediate and adverse prognosis, while both HiDAC regimen and SDMSC can be used prior to transplantation. However, this study has inherited limitations, such as single center, retrospective study, small sample size (especially in the adverse risk group), lack of detailed data on the genetic characteristics of patients at diagnosis and on MRD after consolidation, and failure to compare treatment-related toxicities between regimens. Therefore, prospective randomized multicenter clinical trials are needed to confirm the results of this study.

## Data availability statement

The raw data supporting the conclusions of this article will be made available by the authors, without undue reservation.

## Author contributions

XW and DL collected data, XW wrote the original paper, analyzed data and completed the tables and figures. CZ designed research, performed research, analyzed and interpreted data, critically reviewed the manuscript. All authors contributed to the article and approved the submitted version.
